# Determination of the Critical Voltage for the Observation of Uncoated Wood Samples in Electron Microscopy

**DOI:** 10.3390/ma18020236

**Published:** 2025-01-08

**Authors:** Monika Sarvašová Kvietková, Ondřej Dvořák, Kryštof Kubista, Kristýna Těhníková, Chia-Feng Lin, Dennis Jones

**Affiliations:** 1 Department of Wood Processing and Biomaterials, Faculty of Forestry and Wood Sciences, Czech University of Life Sciences Prague, Kamýcká 1176, 16500 Prague, Czech Republic; 2 Department of Engineering Sciences and Mathematics, Wood Science and Engineering, Luleå University of Technology, Forskargatan 1, 93187 Skellefteå, Sweden; chia-feng.lin@ltu.se; 3 Biotechnical Faculty, University of Ljubljana, Jamnikarjeva 101, 1000 Ljubljana, Slovenia

**Keywords:** electron microscopy (EM), SEM analysis, surface, wood, voltage, density

## Abstract

Electron microscopy (EM) is a key tool for studying the microstructure of wood; however, observing uncoated samples poses a challenge due to surface charging. This study aims to identify the critical voltage that allows for the effective observation of uncoated wood samples without significant loading. As part of the experiment, samples of different wood species were tested, including Acacia (*Robinia pseudoacacia* L.), Oak (*Quercus robur* L.), Maple (*Acer pseudoplatanus* spp.), Ash (*Fraxinus excelsior* L.), Spruce (*Picea abies* (L.) Karst.), Thermowood (Thermal modifed Spruce), Garapa (*Apuleia leiocarpa*), Ipé (*Handroanthus* spp.), Merbau (*Intsia bijuga*), and Massaranduba (*Manilkara* spp.). Several methods were tested for surface preparation for SEM analysis, including the use of a circular saw, a hand milling machine, and a microtome. The results show that the optimal voltage for observing uncoated wood samples varied depending on the wood species. Regarding the selection of wood species and the results obtained, it was found that uncoated samples could be effectively observed. This finding suggests that practical observations can be accelerated and more cost-effective, as all wood species exhibited the required voltage range of 1 kV to 1.6 kV. Additionally, it was determined that using a secondary electron detector was optimal for such observations, as it provided a sufficiently strong signal even at relatively low voltages. Conversely, when using a backscattered electron detector, it was more beneficial to use coated samples to achieve a sufficient signal at higher voltages. This study brings new knowledge that will facilitate further research and applications of electron microscopy in the study of other wood species or wood-based materials.

## 1. Introduction

Electron microscopy is one of the most important techniques for carrying out detailed analyses of materials at the micro and nano level [1,2]. It allows scientists to study structures with a high resolution, which is crucial for many scientific and industrial applications [3]. The most common types of electron microscopes are the transmission electron microscope (TEM) and the scanning electron microscope (SEM), each providing specific advantages for different applications [4,5]. TEMs work by allowing electrons to pass through thin samples and provide information about the internal structure of materials, while SEMs use secondary electrons to produce detailed surface images of samples [2,4]. During the observation of a sample in an electron microscope, the number of electrons emitted from the observed location on the sample is not identical to the number of electrons that strike the sample in the form of an electron beam [3]. In some cases, within a specific range of primary beam energies, the number of electrons emitted from the surface may exceed the number of incident electrons [6]. The charge created by this difference is then dissipated through the conductivity of the sample and is eventually compensated for by the impact of ions from the surrounding environment [2]. Ideally, electron microscopy is conducted on conductive samples, as their ability to dissipate charge prevents sample charging [7], which is crucial for accurate imaging of the observed structures [5]. In the case of non-conductive samples, a simple solution is to coat the sample with a thin conductive layer, typically only a few nanometers thick, that allows the passage of electrons and minimally affects the imaging of the original sample [4,6,7]. However, coating samples with a conductive layer brings several challenges [8], such as being time-consuming, increasing cost [9], and requiring specialized equipment and materials [4,7,8].

In some cases, coating a sample may be undesirable, especially when it is important to preserve the original properties of the sample’s surface [10]. Even a thin conductive layer can alter the final image, potentially obscuring fine details, which could be problematic for certain studies. Therefore, finding optimal conditions for the observation of non-conductive samples without coating has become a key focus in electron microscopy research [11], with researchers actively seeking alternative methods [12]. These include, for example, the use of low-vacuum electron microscopy or the application of small accelerating voltages. Such techniques can help minimize sample charging whilst preserving its original properties, which is particularly important for the study of biological and organic materials such as wood [13]. From the perspective of microstructural investigation, wood presents significant challenges for SEMs [14]. Due to the non-conductivity and high porosity of wood, charging is a major problem [1,2,7]. Additionally, wooden specimens are sensitive to beam damage, especially at higher magnifications [4]. Despite these challenges, the benefits of SEMs are clear, with several research groups demonstrating the potential of SEMs, including work led by Kamdem et al. [15], Sader et al. [16], and Kitin et al. [17].

As already indicated, researchers face specific challenges when studying organic materials such as wood, especially when it comes to uncoated samples. One of the main complications is the charging of the sample, which is caused by the accumulation of electrons in the sample and their uncontrolled discharge [18,19]. This phenomenon can lead to image defects that significantly reduce the quality and interpretability of the acquired images [20]. Intense charging can even cause physical damage to the sample, which is especially problematic for archeological samples and other sensitive organic materials [21]. Defects caused by charging can lead to misinterpretations of the material’s microstructure and properties [18,21]. Thus, finding the critical voltage that minimizes charging and image defects is vital for optimizing observational conditions [22]. The critical voltage is the specific accelerating voltage at which the best compromise between a sufficient image contrast and the minimization of sample charging is achieved [19]. This voltage depends on several factors, including the type of material, its structure, and electrical properties [23]. The size of the charge depends on the energy of the electrons and the number of electrons hitting the sample [24]. The energy of the electrons is related to the accelerating voltage (i.e., high kV = high power), so reducing the accelerating voltage can help reduce the size of the charge. Additionally, the number of electrons hitting the sample is related to several parameters. For example, reducing the number of electrons by changing the aperture size, reducing the current, or narrowing the beam can all contribute to reducing the charge of the sample [25]. A way to observe non-conductive samples in a high-vacuum electron microscope is to find the critical energy at which the number of electrons emitted from the sample equals the number of incident primary electrons. At this critical energy, the sample remains unchanged, allowing for more accurate observations [26,27].

This study aims to find the critical voltage for the observation of uncoated wood samples using an SEM. Specifically, it identifies the voltage at which the best images are obtained with minimal impact from excess charging. It investigates how the various physical properties of wood affect charging and image quality. The study includes the preparation of wooden samples, experiments at different voltage levels, and a systematic assessment of image quality under varying conditions. The study provides a detailed description of the technical provision and experimental steps used to optimize the imaging conditions. The goal of this work is to create optimal conditions for electron microscopy using an SEM for different types of wood, which could serve as a reference for other researchers and experts in this field.

## 2. Material and Methodology

### 2.1. Samples

**Figure 1:**
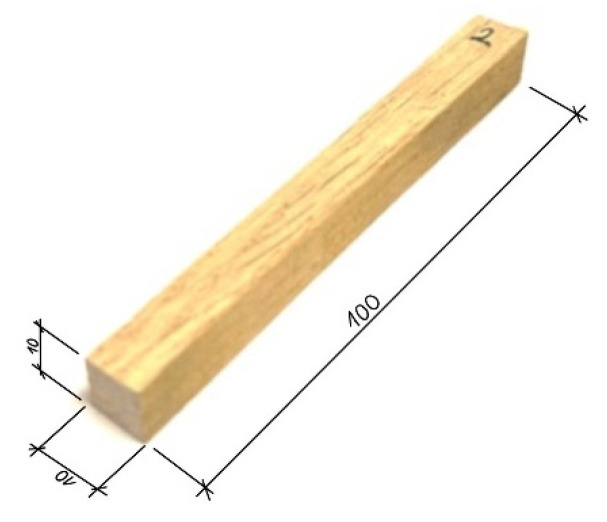
Oak sample (dimensions in millimeters).

Wood samples from both common and exotic species were selected to determine whether the specific type of wood affected the observations in the SEM (Thermo Fisher Scientific Inc., Waltham, MA, USA). The selected samples were Acacia (*Robinia pseudoacacia* L.), Oak (*Quercus robur* L.), Maple (*Acer pseudoplatanus* spp.), Ash (*Fraxinus excelsior* L.), Spruce (*Picea abies* (L.) Karst.), Thermowood (Thermal modifed Spruce), Garapa (*Apuleia leiocarpa*), Ipé (*Handroanthus* spp.), Merbau (*Intsia bijuga*), and Massaranduba (*Manilkara* spp.). The choice of classic spruce and Thermowood™ helped to assess whether thermal treatment of the wood affected the structural observations. Specimens with dimensions of approx. 10 × 10 × 100 mm (Tangential × Radial × Longitudinal) were cut from each species (Figure 1). A frit was used to prepare samples for observation along and perpendicular to the fibers. The aim was to obtain dust-free samples with an intact wood structure. Ensuring that the specimen was dry was essential to prevent potential chamber contamination and sample damage. In a vacuous environment, moisture within the specimen could evaporate along with extractives, potentially contaminating the chamber and damaging the sample.

**Figure 2:**
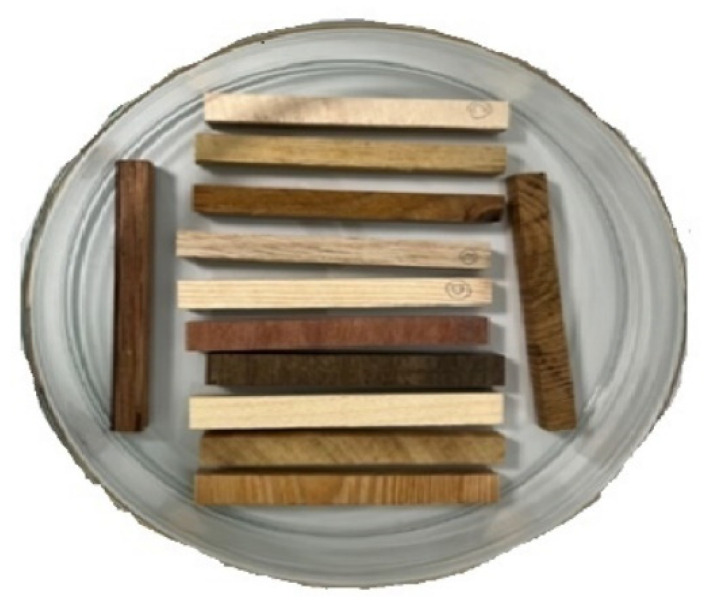
Preparation of samples in a Petri dish.

The samples were weighed and placed in Petri dishes before being heated in an oven at 103 °C (see Figure 2). After four hours, the samples were weighed again and then returned to the oven for an additional two hours before being weighed to see if the weight was constant. By the third weighing, the weight of the samples remained unchanged from the second weighing. Based on the determined weights, the original moisture content of each sample was calculated according to Equation (1) and the results are presented in Table 1.

(1) $$w=\frac{M_{V}}{M_{SD}}\times 100$$

*w*—Wood humidity.

*M_V_*—Weight of water.

*M_SD_*—Weight of completely dry wood.

The densities of the samples were calculated using Equation (2) to determine any potential effect on the observation of samples in the electron microscope (see Table 2).

(2) $$\rho =\frac{m}{V}\times 100$$

*m*—Sample weight.

*V*—Sample volume.

**Figure 3:**
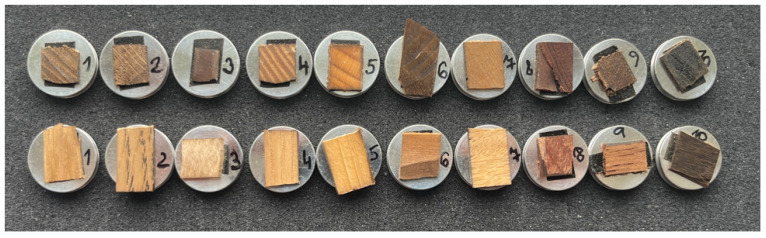
Final set of samples for SEM observation.

During the preparation of the semi-finished products, the surfaces of the specimens appeared smooth and clean after using a circular saw or a hand milling machine, respectively. Therefore, samples for the SEM were cut to a thickness of approx. 1 × 4 × 4 mm using a table band saw. These samples were then attached to stubs with the machined side resulting from the bandsaw cut and placed into the SEM. An additional method of preparation used a microtome. After plasticizing the semi-finished products, blocks of each wood species with dimensions of approx. 10 × 10 × 10 mm were clamped in a microtome, and their surfaces were cut with a sharp blade to obtain an intact, smooth transverse and longitudinal surface. Once the observational areas were prepared on the microtome, careful drying was necessary due to the plasticization of the samples. Thus, the samples were first air-dried and then placed in an airtight box with silica gel for further drying. The samples prepared in this way were then attached to stubs of the ground surface using double-sided adhesive carbon tape (Figure 3). An optical microscope (Thermofisher model Quattro, Waltham, MA, USA) was used to check for drying damage (e.g., cracks) and to ensure the surfaces appeared smooth and free of debris (e.g., lint and dirt).

**Figure 4:**
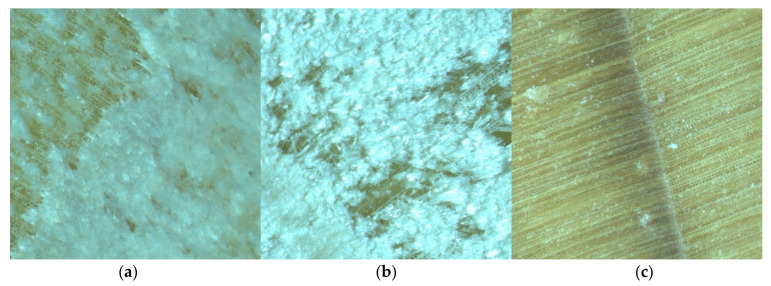
Sample of spruce after circular saw (**a**), milling machine, (**b**) and microtome (**c**).

A more detailed comparison of the surfaces of the samples was undertaken using optical microscopy at 50× magnification. Figure 4a–c show examples of Spruce wood under different sample preparation methods: (a) the surface after circular sawing, (b) the surface after milling machine, and (c) the surface after microtome processing.

### 2.2. Electron Microscopy

A ThermoFisher Scientific Quattro environmental scanning electron microscope (ESEM) (Waltham, MA, USA), equipped with a field emission gun (FEG), was used to study wood samples without further surface coating with a conductive layer. This model is ideal for a wide range of applications where flexibility is required, as is the case with wood [28]. A working distance (WD) of about 7 mm was set for the analysis.

### 2.3. Process/Method

**Figure 5:**
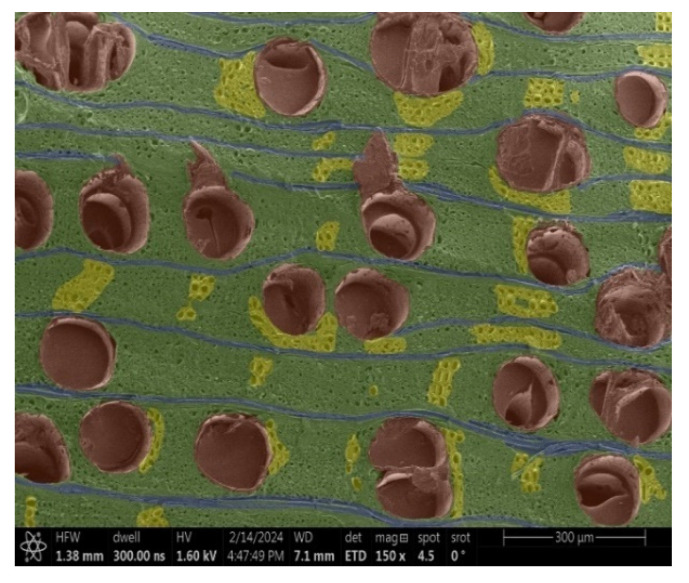
Image of Garapa colored in MIB.

The investigation of a suitable voltage at which the sample will not charge was carried out by gradually adjusting the voltage in steps of 0.1 kV, starting from the lowest setting. At 0.1 kV, no signal was observed, while at 0.3 kV, the wood became observable, though the signal was weak. At 0.5 kV, the signal was sufficient for observation, the sample did not charge, and its structure was visible. At 1.0 kV, the sample was also not charged, the depth of field improved, and the structure was very clearly visible. The same result was achieved at accelerating voltages of 1.1 kV, 1.2 kV, and 1.3 kV, with each 0.1 kV increase further enhancing the depth of field, sharpness, and resolution of the image. However, at 1.8 kV, all samples began to charge. When the sample surface became charged, it was necessary to move the observation point to an area that was not charged. If the entire surface was charged, the vacuum in the chamber had to be reduced by admitting air to discharge the sample. After discharging, the vacuum was re-established for observation. During the observation, images were monitored and evaluated based on color and the characteristic charging artifacts created. The state of charge was recorded on the record sheet. Each sample was observed at ten different locations, using four samples for each wood species: two for observations parallel to the grain and two for observations perpendicular to the grain. Following this, the images were colored according to the individual anatomical parts of the selected wood species (Figure 5) using a post-process technique (for example, green—wood; brown—big pores; yellow—small pores; blue—microfibrils). The MIB (microscopy image browser “ELIXIR Bio-tools, Helsinki, Finland”) program was chosen for this coloring.

The resulting surface images were compared with images from the database within Mamoňová’s 2019 study [29].

## 3. Results and Discussion

**Figure 6:**
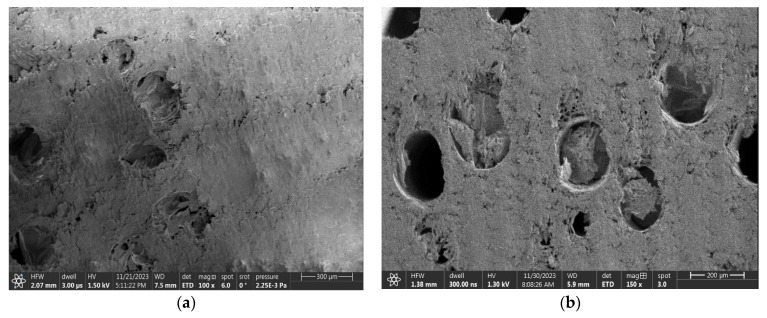
SEM images of the oak surface after using a circular saw (**a**) and after processing with a milling cutter (**b**).

It was found that the surfaces prepared using the circular saw were not smooth enough for SEM analysis. The wood structure was not visible because the surfaces were fibrous, with the fibers either bent or rubbed into the sample (Figure 6a). Thus, this sample preparation method was considered unsuitable for the SEM analysis. At the same time, charging from the tips of the protruding fibers was visible even at lower voltages. In contrast, the milled surfaces appeared smooth and shiny to the naked eye. However, when these samples were examined under an optical microscope, the results varied. In some wood species, the structure appeared intact, while in others it was clear that this preparation method was also not suitable. This was further confirmed by the SEM analysis (Figure 6b), which led to the conclusion that this preparation method was also unsatisfactory. The microtome method, however, provided a superior image quality, as supported by studies such as those by Tokarev et al., where additional surface modification was applied [30]. Jansen et al. [31] and Balzano et al. [32] also found that softening the surface before making a cut was beneficial. Despite these advancements, it was evident that achieving an ideal surface through simple cutting or grinding remains a challenge.

Based on the visual evaluation, no specific accelerating voltage corresponded to the critical energy. However, there were voltage ranges where the samples did not exhibit charging during observation, and the images were free from charging artifacts. Within these ranges, the signal was strong enough to clearly reveal the wood’s structure. The observation results are summarized in Table 3. The table shows that the voltage of 0.5 kV was the lowest viable voltage for monitoring across all wood species, regardless of the cut. While the optimal voltage was the same, the maximum voltage varied between species.

Table 4 reveals that the type of wood alone was not the sole factor affecting the maximum possible observation voltage. This was evident from the fact that the same wood species showed different critical voltages when observed along the grain versus perpendicular to it. Whilst the wood type can be a contributing factor, it was not the determining one.

Investigating the correlation between density and charging behavior showed that density was not likely to be the only factor influencing the results. Although density may contribute to some extent, samples from the same wood species, cut lengthwise and crosswise (thus having identical density), did not show the same highest applicable voltage. From this, it can be concluded that the charging behavior of the wood samples may be more influenced by their structure in the direction of observation than by density alone. The structure of wood includes various microstructural elements, such as tracheids, fibers, vessels, and their distribution, which can significantly influence the behavior of electrons on the surface of the sample. These elements can cause different levels of charging depending on the orientation of the cut and how electrons are scattered and absorbed within the material.

Furthermore, it was necessary to consider that different types of wood can have different chemical compositions and moisture contents, which can also affect the charging behavior. For example, wood with a higher content of resin or other organic substances may show different electrical properties compared to wood with lower levels of these components. Therefore, it is evident that the issue of charging wood samples during electron microscopy is complex and influenced by multiple factors. Beyond wood type and density, factors such as microstructure, chemical composition, and moisture content also play roles.

Based on structural similarity among woody plants, the following groups could be categorized:

Spruce and Thermowood™: These woods have a uniform structure of spring and summer tracheids.

Acacia, Oak, Garapa, Ash, Massaranduba and Merbau: These species are characterized by a structure with relatively large vessels surrounded by smaller calyxes and uniform libriform fibers interwoven with parenchymal cells.

Maple: This wood features significantly smaller vessels, resulting in a more uniform structure compared to the species mentioned above.

Ipé: This wood has a very smooth structure, with only the lumens of isolated vessels visible.

No clear dependence was evident between the highest applicable voltage and structure. Based on the results of the comparison of structure similarity and all the above comparisons, the composition of woody species was proposed as another possible factor influencing charging, mainly in terms of extractive substances, their composition, and quantity. Thus, the following observations could be made on the required voltage in terms of species and their relevant structures:

✓ Spruce and Thermowood™:
  - Structure: Uniform tracheids that form a homogeneous network.
  - Voltage: Due to the uniform structure, a uniform distribution of electric charge is assumed, but practical tests have shown that this may not be a decisive factor.
✓ Acacia, Oak, Garapa, Ash, Massaranduba and Merbau:
  - Structure: A combination of large vessels and smaller vessels surrounded by libriform fibers and parenchymal cells.
  - Voltage: The heterogeneous structure can cause uneven charging, which can lead to image artifacts during observation.
✓ Maple:
  - Structure: Smaller and more uniform vessels compared to other woods.
  - Voltage: A more uniform structure provides more stable electrical properties when observed, but again a direct relationship between structure and charge was not found.
✓ Ipé:
  - Structure: Extremely smooth, with minimal visible veins.
  - Voltage: It was hypothesized that the smooth structure might minimize charging, but the results were inconclusive.

The chemical composition of the wood species, especially the content of extractive substances, can play a crucial role in their electrical properties. These substances can affect the conductivity of the wood and thus its charging behavior under electron microscopy. Furthermore, the presence of extractives and their composition varies between individual trees and also depends on the direction of observation. The amount of resin is determined by where the cut is made and can be more significant in cross-sections or longitudinal sections. This could affect the observational voltage and charging resistance. In the cross-section, where there was a high resin content on the surface, the resistance to charging was minimal. On the other hand, in the longitudinal section, where there was a smaller amount of cut fibers and therefore a smaller amount of resin on the surface, the voltage required was slightly higher.

**Figure 7:**
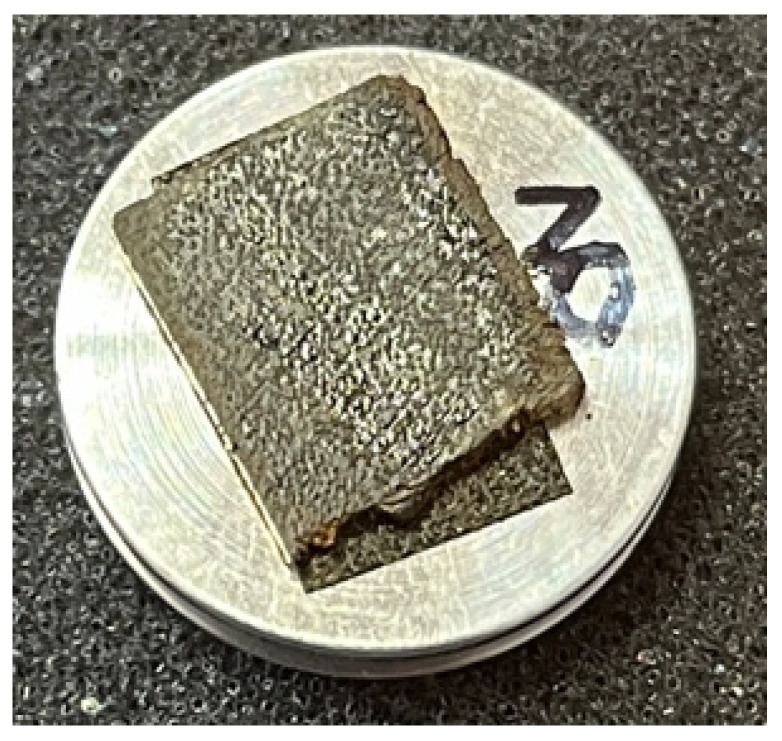
Cross-section of Ipé with blown resin.

**Figure 8:**
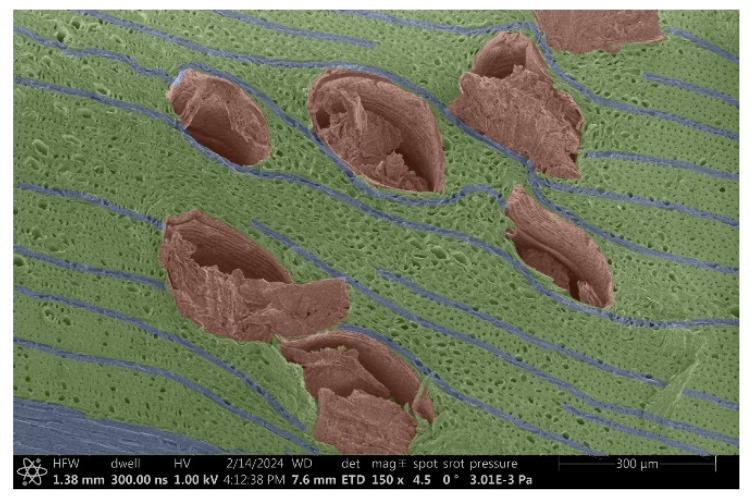
Sample of Oak.

**Figure 9:**
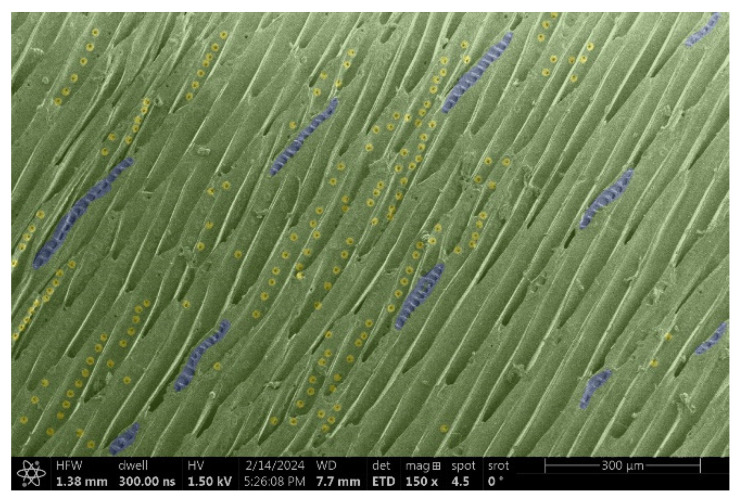
Sample of Spruce.

Ipé wood produced a relatively large amount of resin during the preparation of the cross-sectional sample (Figure 7). The surface was very sticky, and these samples showed the lowest resistance to charging. On the other hand, the sample in the longitudinal section did not have resin exudation on the surface, but it was visible in the section within individual fibers. Several authors, such as Shan et al. [33], have used delignification and water extraction to observe porous surfaces, such as wood. With this modification, it was possible to increase the electrical capacity and subsequent image quality. In previous work, Silveira et al. [34] monitored the properties of wood cells using SEM analysis. They agreed that it was essential that wood samples were adequately prepared before electron microscopy, which may include drying or coating with a thin layer of conductive material to prevent charging the sample with electrons. Figure 5, Figure 8 and Figure 9 show the differences in the images of the coated and uncoated samples.

In Figure 5, large pores are visible in both images. The uncoated sample even shows the structure inside the pores (vessel). The coated surface better shows the small pores and also makes the boundaries between different parts of the wood more visible.

Figure 8 confirms what was seen in Figure 5, where there was visible thinning in parts of the uncoated sample, even in the close-up image, compared to the sample of Spruce shown in Figure 9.

From the images in Table 5, it can be seen that a high-quality image can be created even without coating. The determination of the critical voltage for observing uncoated wood samples was vital. In electron microscopy, it is essential to ensure that samples are not damaged during the scanning process. In the case of wood, which is an anisotropic material, it was necessary to determine the critical voltage by using various methods, including experimental tests and theoretical calculations, as was the case in the studies of, e.g., Wentzel et al. [35] and Setyawan et al. [36]. Among others, the critical energy in non-conductive samples was determined by Chen et al. [37] and Shan et al. [38], who described a procedure to detect the critical energy using a computer-controlled scanning electron microscope. The results from this study can help find an effective solution to this problem. Van Huis and Friedrich [39] introduced the basic concepts of electron microscopy (EM). The result was a comprehensive set of tools for characterizing nanoparticles’ size, three-dimensional shape, composition, or crystal structure. In this study, they also considered options that confirm our research direction. Müller et al. [40] considered using a higher voltage, which may be suitable for materials other than wood. The results showed that this method can reduce the amount of time associated with sample preparation for SEM.

## 4. Conclusions

Our research shows that the direction of the cut, the presence of resin, and the structure of the wood species influence the quality of wood observations performed by electron microscopy and necessitate an individual approach to setting parameters. Furthermore, it was found that optimization of sample preparation, such as surface resin removal, can significantly improve image quality and minimize charging. In summary, we made the following conclusions:

(1) The observational results showed that the charging of wood samples under electron microscopy was influenced not only by the type of wood but also by the direction of the cut and the presence of extractive substances, such as resin. It was noted that Ipé released a large amount of resin in the transverse section, resulting in minimal resistance to charging, whilst in the longitudinal section, the resin was present inside the fibers and the sample showed a higher resistance, supporting the hypothesis of the effect of extractives on charging.

(2) Another important finding was that neither the wood density nor the wood species were the only factors influencing the highest possible observation voltage. It turned out that the structure of the wood and the presence of extractive substances played a key role. This suggested that the complex interaction of these factors needs to be taken into account to achieve optimal results in the electron microscopy of wood.

(3) The analysis also revealed that, when preparing the samples, it was necessary to pay extra attention to the direction of the cut and the presence of resin on the surface. Samples with more surface resin charged more, which degraded the quality of the images. This finding suggested that optimizing sample preparation, for example, by removing or reducing surface resin, could significantly improve the observational results.

(4) In addition, it was found that when observing different wood species, the accelerating voltage needed to be individually adjusted to minimize charging and maximize image quality. This variability between samples of the same species of wood but different cutting directions highlighted the need for a flexible approach when setting electron microscopy parameters.

Overall, the results indicated that improving the electron microscopy of wood was necessary by using the following strategies:

(a) Thoroughly analyzing and optimizing the preparation of samples related to the direction of the cut and the presence of extractive substances.

(b) Individually adjusting the acceleration voltage for different samples and cutting directions.

(c) Carrying out further research focused on the chemical composition of extractive substances and their effect on the electrical properties of wood.

These recommendations could lead to a significant improvement in the quality and reliability of electron microscopy results for wood samples, which would have a positive impact on a wide range of applications in the field of materials research and industry.

## Figures and Tables

**Table 1:** Trend of weight changes during sample drying.

Sample Number	Type of Wood	Weight Before Drying [g]	Weight After 4 h [g]	Weight After 6 h [g]	Weight of Water [g]	Original Humidity [%]
1	Acacia	7.910	7.420	7.420	0.490	6.60
2	Oak	6.980	6.390	6.390	0.590	9.23
3	Maple	7.715	7.035	7.035	0.680	9.67
4	Ash	8.770	8.005	8.005	0.765	9.56
5	Spruce	5.015	4.540	4.540	0.475	10.46
6	Thermowood™	5.050	4.820	4.820	0.230	4.77
7	Garapa	8.725	8.015	8.015	0.710	8.86
8	Massaranduba	12.210	11.165	11.165	1.045	9.36
9	Merbau	8.615	7.915	7.915	0.700	8.84
10	Ipé	14.370	13.210	13.210	1.160	8.78

**Table 2:** Calculation of the density of individual samples.

Sample Number	Type of Wood	Width [cm]	Height [cm]	Length [cm]	Volume [cm^3^]	Weight After 6 h [g]	Density [g/cm^3^]
1	Acacia	0.943	1.01	10.123	9.641	7.420	0.770
2	Oak	1.064	1.09	10.118	11.734	6.390	0.545
3	Maple	1.031	1.072	9.874	10.913	7.035	0.645
4	Ash	1.01	1.078	10.142	11.042	8.005	0.725
5	Spruce	1.027	1.197	10.14	12.465	4.540	0.364
6	Thermowood™	1.051	1.079	9.61	10.898	4.820	0.442
7	Garapa	1.073	1.039	9.875	11.009	8.015	0.728
8	Massaranduba	1.082	1.029	9.764	10.871	11.165	1.027
9	Merbau	1.04	1.019	10.151	10.758	7.915	0.736
10	Ipé	1.199	1.041	9.718	12.130	13.210	1.089

**Table 3:** Monitored voltage range.

Number	Wood	Fiber Direction	Voltage Range
1	Acacia	parallel to the fibers	0.5–1.4 kV
		perpendicular to the fibers	0.5–1.4 kV
2	Oak	parallel to the fibers	0.5–1.5 kV
		perpendicular to the fibers	0.5–1.6 kV
3	Maple	parallel to the fibers	0.5–1.6 kV
		perpendicular to the fibers	0.5–1.5 kV
4	Ash	parallel to the fibers	0.5–1.4 kV
		perpendicular to the fibers	0.5–1.6 kV
5	Spruce	parallel to the fibers	0.5–1.5 kV
		perpendicular to the fibers	0.5–1.4 kV
6	Thermowood™	parallel to the fibers	0.5–1.3 kV
		perpendicular to the fibers	0.5–1.4 kV
7	Garapa	parallel to the fibers	0.5–1.7 kV
		perpendicular to the fibers	0.5–1.6 kV
8	Massaranduba	parallel to the fibers	0.5–1.6 kV
		perpendicular to the fibers	0.5–1.3 kV
9	Merbau	parallel to the fibers	0.5–1.3 kV
		perpendicular to the fibers	0.5–1.5 kV
10	Ipé	parallel to the fibers	0.5–0.7 kV
		perpendicular to the fibers	0.5–1.2 kV

**Table 4:** Sample charge results sorted by density.

		Voltage [kV]									
Type of Wood	Density [g/cm^3^]	0.5	1.0	1.1	1.2	1.3	1.4	1.5	1.6	1.7	1.8
Spruce	0.364	x	x	x	x	x	x	x	Yes		
Spruce cross	0.364	x	x	x	x	x	x	Yes			
Thermowood™	0.442	x	x	x	x	x	Yes				
Thermowood™ cross	0.442	x	x	x	x	x	x	Yes			
Oak	0.545	x	x	x	x	x	x	x	Yes		
Oak cross	0.545	x	x	x	x	x	x	x	x	Yes	
Maple	0.645	x	x	x	x	x	x	x	x	Yes	
Maple cross	0.645	x	x	x	x	x	x	x	Yes		
Ash	0.725	x	x	x	x	x	x	Yes			
Ash cross	0.725	x	x	x	x	x	x	x	x	Yes	
Garapa	0.728	x	x	x	x	x	x	x	x	x	Yes
Garapa cross	0.728	x	x	x	x	x	x	x	x	Yes	
Merbau	0.736	x	x	x	x	x	Yes				
Merbau cross	0.736	x	x	x	x	x	x	x	Yes		
Acacia	0.770	x	x	x	x	x	x	Yes			
Acacia cross	0.770	x	x	x	x	x	x	Yes			
Massaranduba	1.027	x	x	x	x	x	x	x	x	Yes	
Massaranduba cross	1.027	x	x	x	x	x	Yes				
Ipé	1.089	x	Yes								
Ipé cross	1.089	x	x	x	x	Yes					

Note: insufficient voltage marked with a cross, the highest applicable voltage marked with the word yes.

**Table 5:** General overview of samples.

Type of Wood	Cross	Axial
Acacia	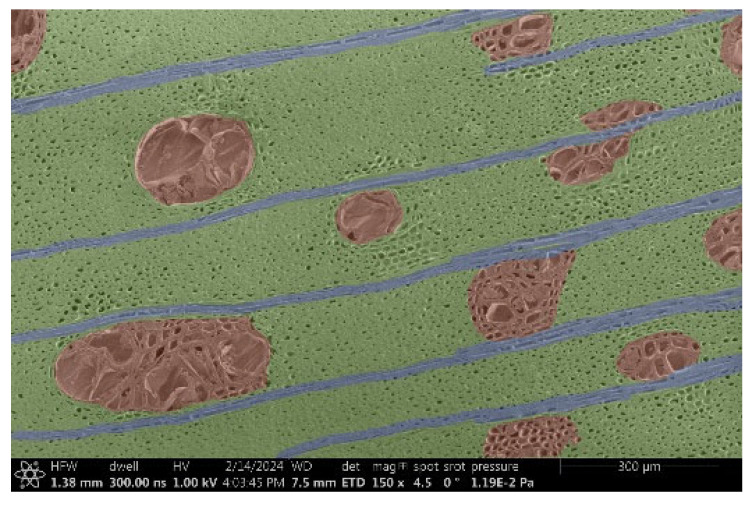	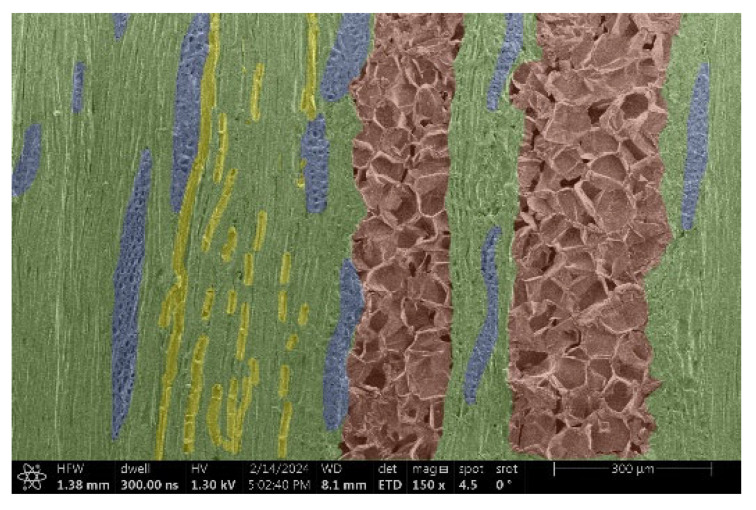
Oak	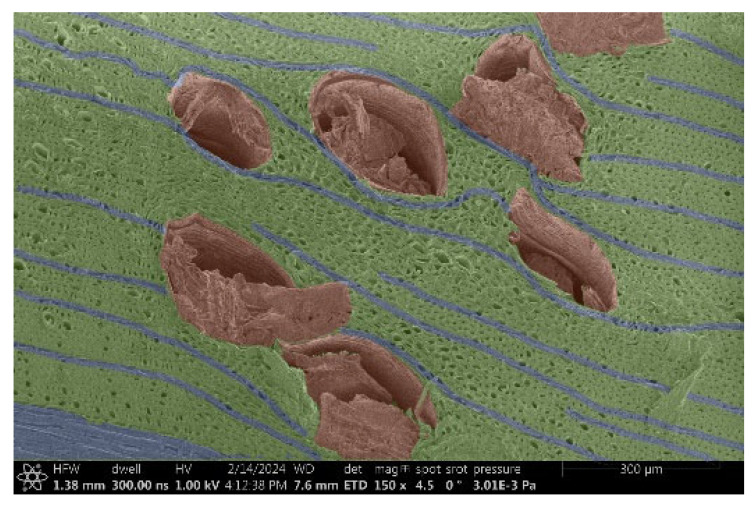	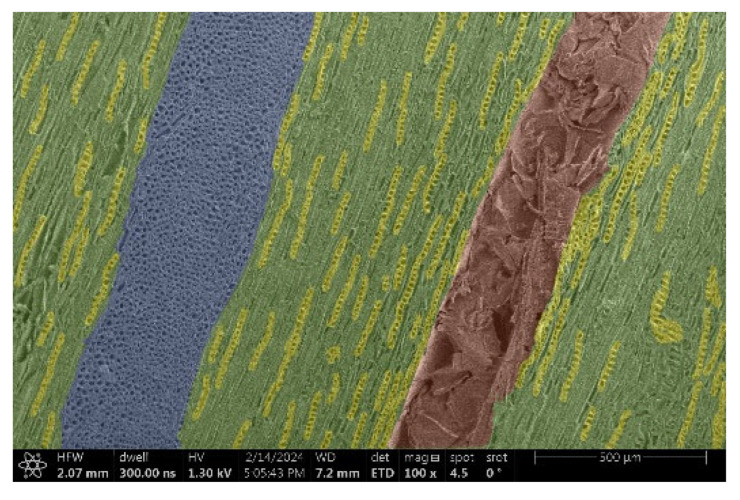
Maple	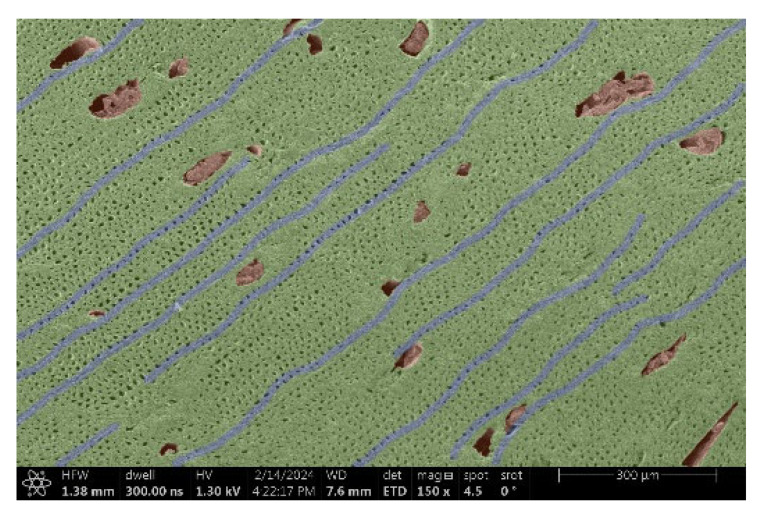	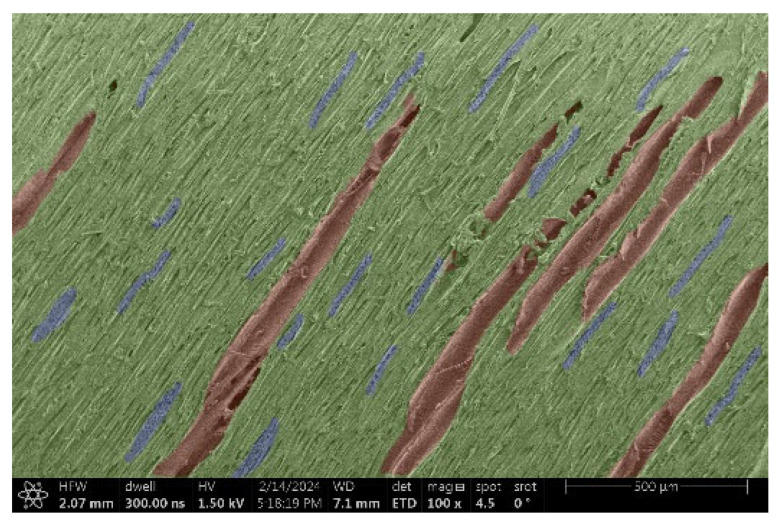
Ash	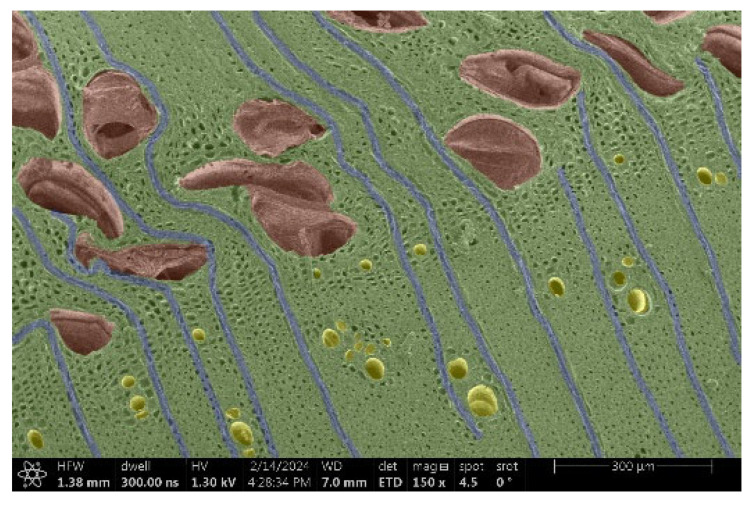	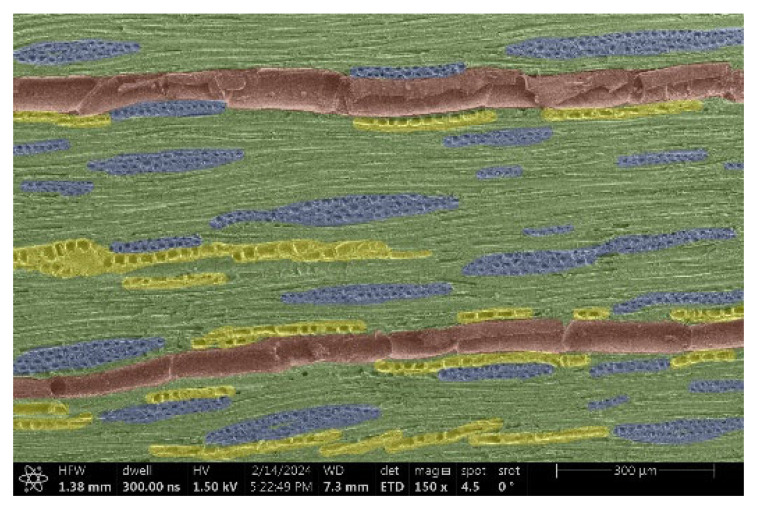
Spruce	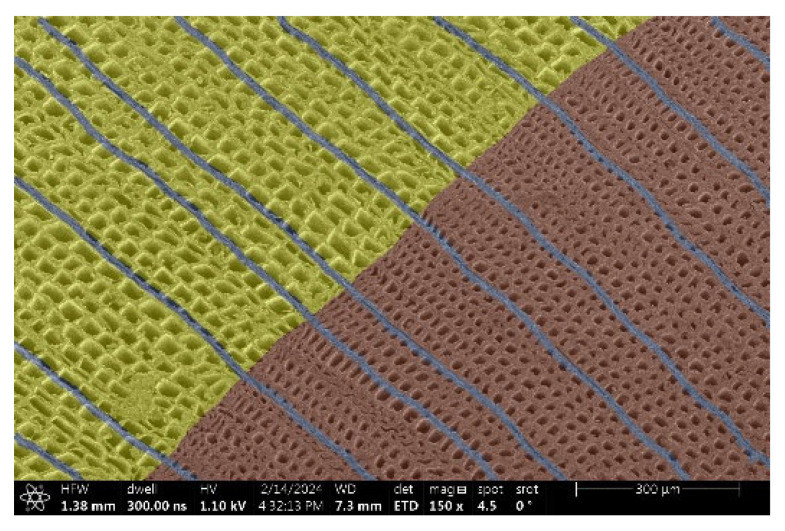	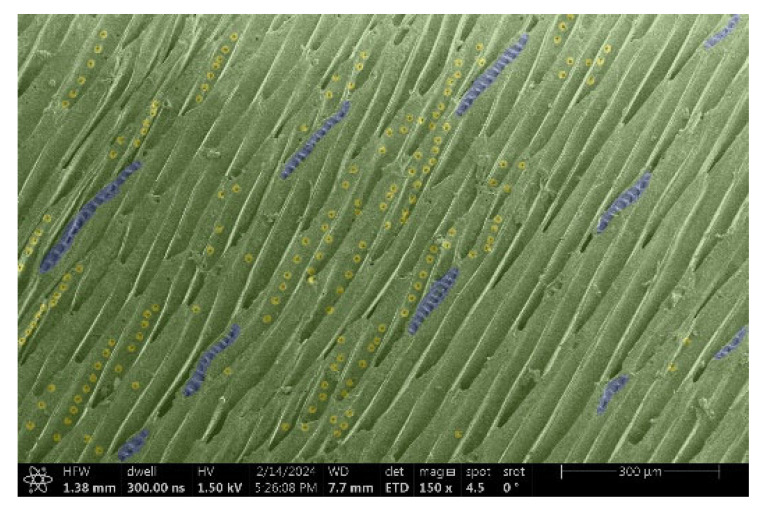
Thermowood™	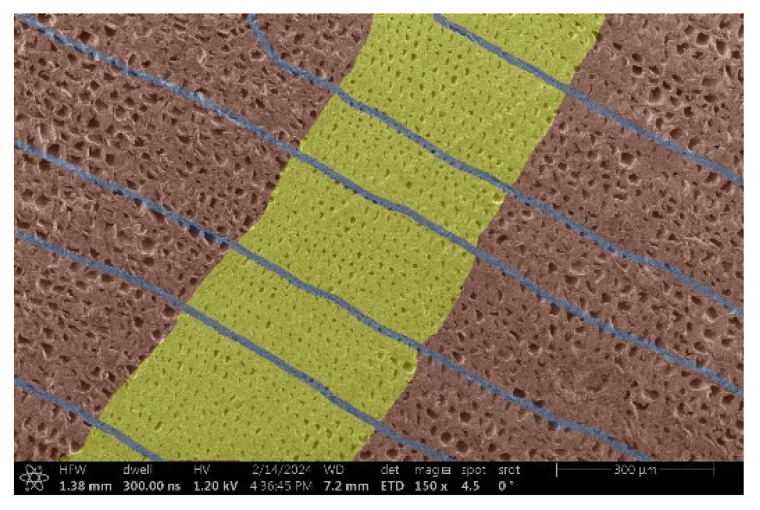	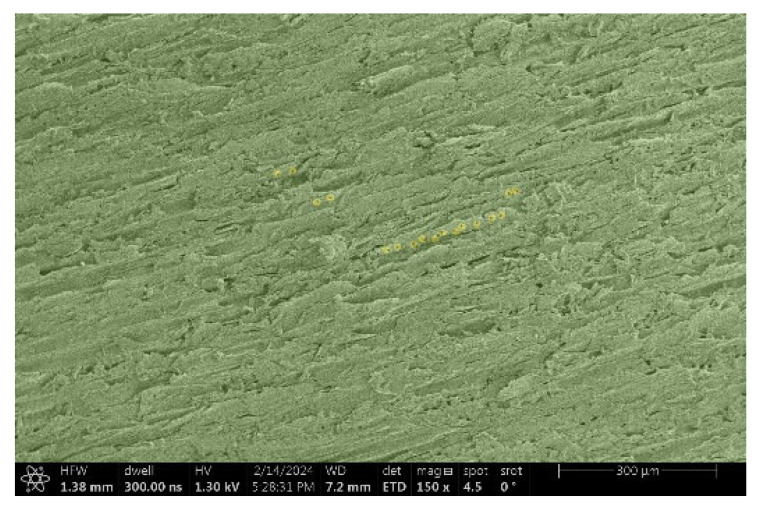
Garapa	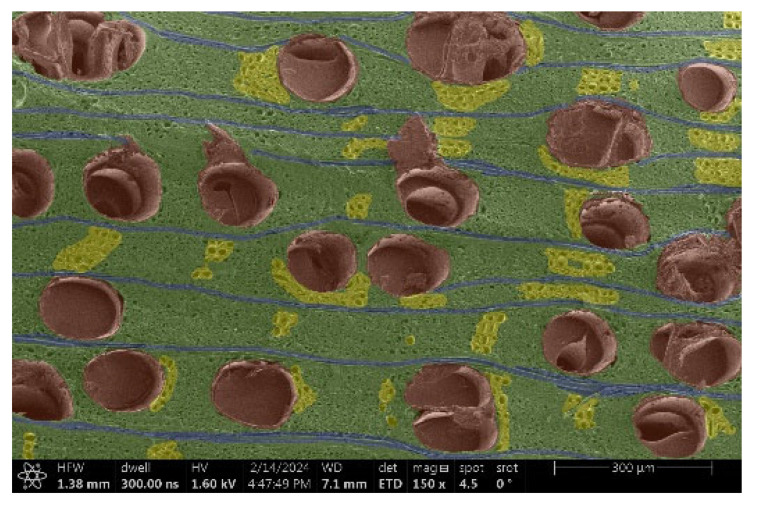	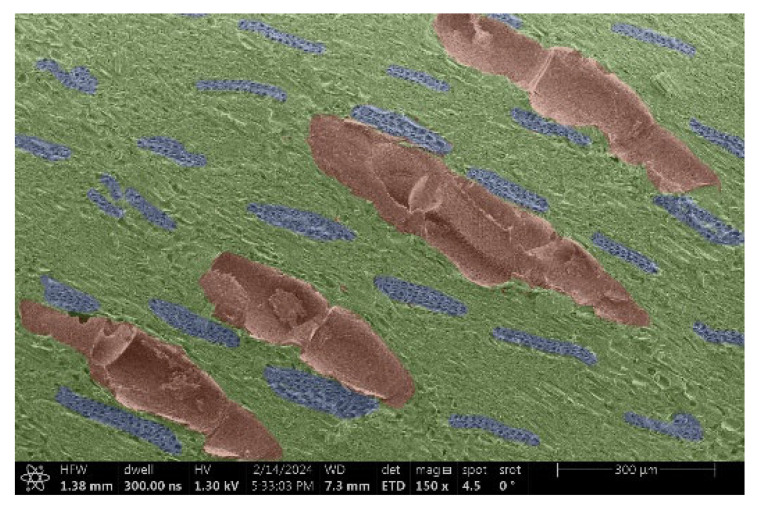
Massaranduba	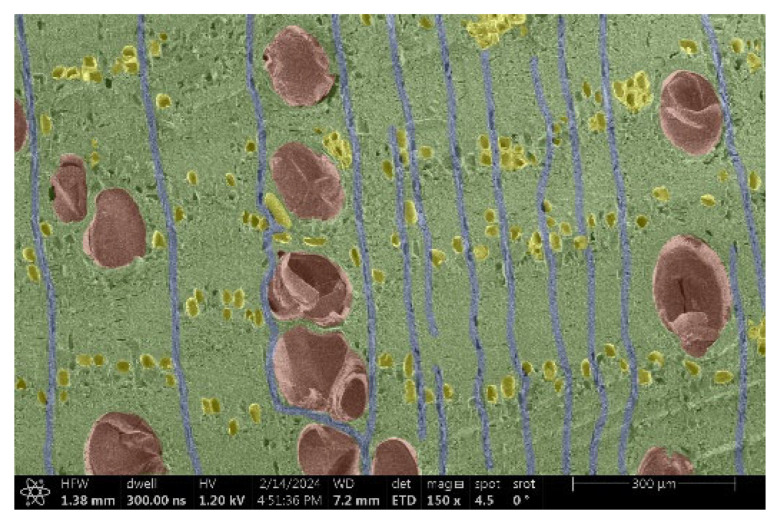	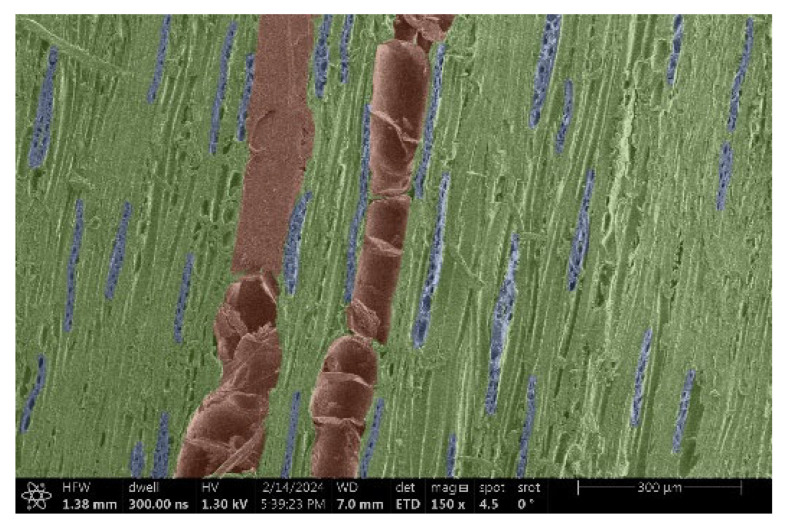
Merbau	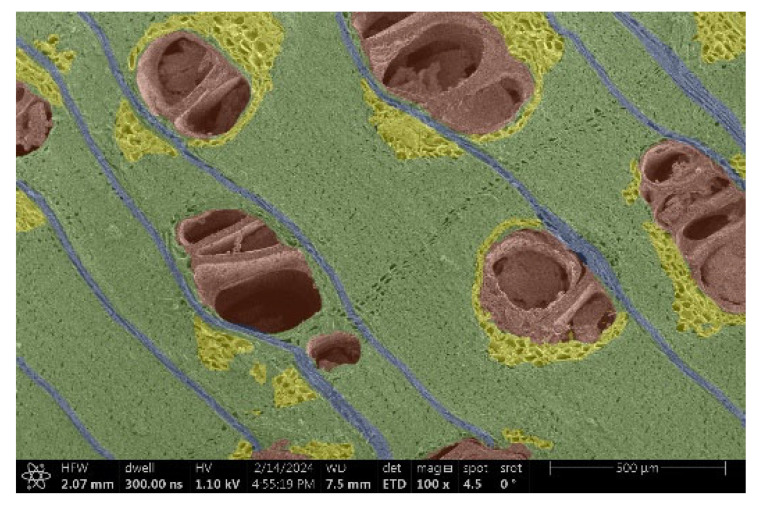	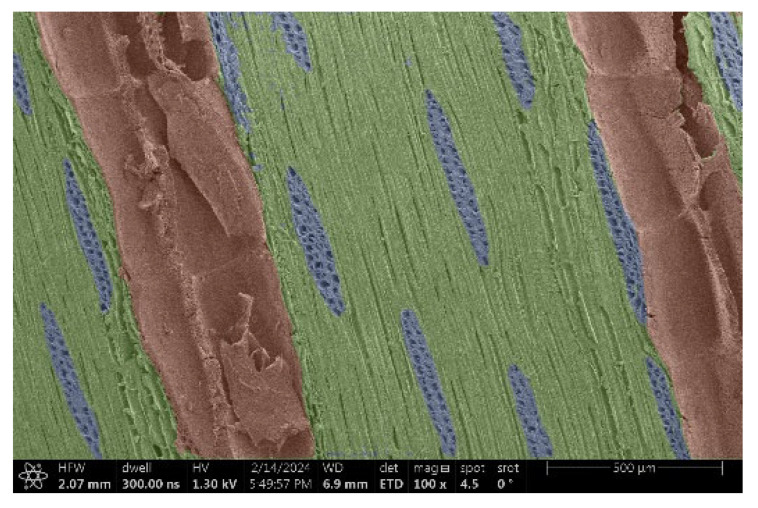
Ipé	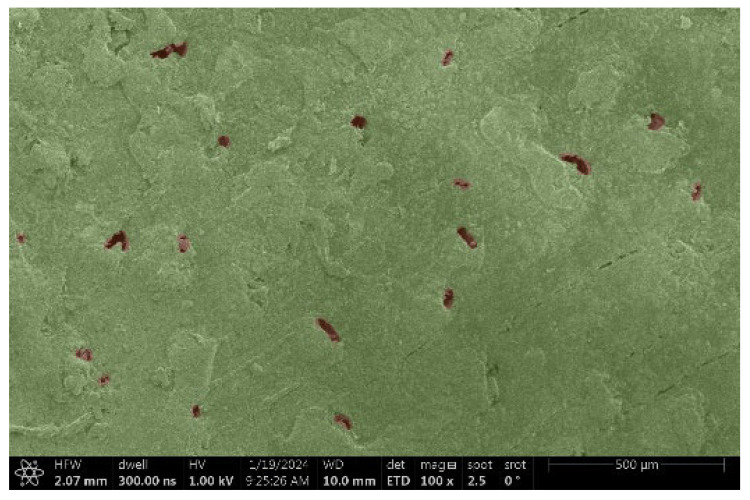	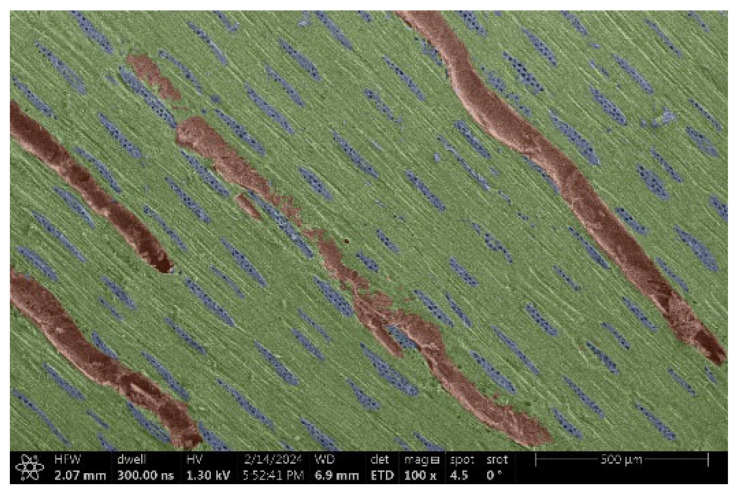

## Data Availability

The original contributions presented in the study are included in the article, further inquiries can be directed to the corresponding authors.
